# Dual activation of Hedgehog and Wnt/β-catenin signaling pathway caused by downregulation of SUFU targeted by miRNA-150 in human gastric cancer

**DOI:** 10.18632/aging.202895

**Published:** 2021-04-12

**Authors:** Yin Peng, Xiaojing Zhang, Huijuan Lin, Shiqi Deng, Ying Qin, Jieqiong He, Fan Hu, Xiaohui Zhu, Xianling Feng, Jian Wang, Yanjie Wei, Xinmin Fan, Huan Lin, Hassan Ashktorab, Duane Smoot, Yansi Lv, Song Li, Stephen J. Meltzer, Zhe Jin

**Affiliations:** 1 Guangdong Key Laboratory for Genome Stability and Disease Prevention, Department of Pathology, Shenzhen University School of Medicine, Shenzhen 518060, Guangdong, P.R. China; 2 Guangdong Provincial Key Laboratory of Regional Immunity and Diseases, Department of Pathology, Health Science Center, Shenzhen University, Shenzhen 518060, Guangdong, P.R. China; 3 Department of Pathology, Guangdong Province Key Laboratory of Molecular Oncologic Pathology, Guangzhou 510515, Guangdong, P.R. China; 4 Department of Ultrasound, Guangdong Women and Children Hospital, Guangzhou 510000, Guangdong, P.R. China; 5 Department of Pathology and Pathophysiology, Guangzhou Medical University, Guangzhou 510000, Guangdong, P.R. China; 6 Department of Gastrointestinal Surgery, The First Affiliated Hospital of Shenzhen University, Shenzhen 518000, Guangdong, P.R. China; 7 Center for High Performance Computing, Shenzhen Institutes of Advanced Technology, Shenzhen 518000, Guangdong, P.R. China; 8 Department of Vascular Surgery, The First Affiliated Hospital of Shenzhen University, Shenzhen 518060, Guangdong, P.R. China; 9 Department of Medicine and Cancer Center, Howard University, College of Medicine, Washington, DC 20060, USA; 10 Department of Medicine, Meharry Medical Center, Nashville, TN 37208, USA; 11 Shenzhen Science and Technology Development Exchange Center, Shenzhen 518060, Guangdong, P.R. China; 12 Department of Medicine, GI Division, Johns Hopkins University School of Medicine and Sidney Kimmel Comprehensive Cancer Center, Baltimore, MD 21287, USA

**Keywords:** miRNA-150, SUFU, Wnt/β-catenin, hedgehog, EMT

## Abstract

Mounting evidence has shown that miRNA-150 expression is upregulated in gastric cancer (GC) and is associated with gastric carcinogenesis, but the underlying oncogenic mechanism remains elusive. Here, we discovered that miRNA-150 targets the tumor suppressor SUFU to promote cell proliferation, migration, and the epithelial–mesenchymal transition (EMT) via the dual activation of Hedgehog (Hh) and Wnt signaling. MiRNA-150 was highly expressed in GC tissues and cell lines, and the level of this miRNA was negatively related to that of SUFU. In addition, both the miRNA-150 and SUFU levels were associated with tumor differentiation. Furthermore, miRNA-150 activated GC cell proliferation and migration *in vitro*. We found that miRNA-150 inhibitors repressed not only Wnt signaling by promoting cytoplasmic β-catenin localization, but also repressed Hh signaling and EMT. MiRNA-150 inhibition also resulted in significant tumor volume reductions *in vivo*, suggesting the potential application of miRNA-150 inhibitors in GC therapy. The expression of genes downstream of Hh and Wnt signaling was also reduced in tumors treated with miRNA-150 inhibitors. Notably, anti-SUFU siRNAs rescued the inhibitory effects of miRNA-150 inhibitors on Wnt signaling, Hh activation, EMT, cell proliferation, cell migration, and colony formation. Taken together, these findings indicate that miRNA-150 is oncogenic and promotes GC cell proliferation, migration, and EMT by activating Wnt and Hh signaling via the suppression of SUFU expression.

## INTRODUCTION

Gastric cancer (GC) is a common neoplastic disease worldwide [1] and is highly prevalent in China. In fact, nearly half of GC patients worldwide are Chinese [2]. Late-stage GC is known to have a very poor prognosis as opposed to early-stage GC, which has a much better prognostic outlook. Study of the carcinogenic mechanisms underlying GC will help to optimize the treatment strategies and improve the overall survival of GC patients.

Previous studies have shown that miRNA-150 is overexpressed in GC cell lines and tissues [3, 4]. Ectopic expression of miRNA-150 has been found to promote the tumorigenesis and proliferation of GC cells. A high level of miRNA-150 is associated with a shorter time-to-progression and shorter overall survival in GC patients undergoing palliative chemotherapy [5]. One mechanistic study reported that *Helicobacter pylori* (HP) infection significantly increased the expression of several miRNAs, including miRNA-150, and consequently downregulated the expression of all selected DNA mismatch repair (MMR) genes [6]. MiRNA-150 expression was shown to be dysregulated in GC, but the underlying oncogenic mechanism involving this miRNA remains elusive.

Wnt/β-catenin signaling is known to be involved in gastric tumorigenesis [7–12]. Accumulating data also suggest a correlation between miRNA dysregulation and aberrant Wnt/β-catenin signaling activation during cancer progression. We previously showed that miRNA-194 induces the activation of the Wnt/β-catenin signaling pathway by inhibiting the expression of SUFU [13]. SUFU is a well-known negative regulator of the Hedgehog (Hh) signaling pathway. Aberrant activation of Hh signaling is often observed in GC. Increased expression of the Shh, Smo, and Ptch1 receptors has also been observed in GC [14, 15]. Furthermore, emerging evidence has shown that excessive Shh expression is associated with a poor prognosis in GC patients [16, 17]. These reports highlight the importance of the Hh signaling pathway in gastric carcinogenesis and progression, but the regulation of the Hh signaling pathway by miRNA and the crosstalk between the Wnt and Hh signaling pathways have not yet been well studied.

In this study, we found that SUFU is targeted by miRNA-150. The expression level of miRNA-150 was significantly higher in GC tissues than in non-neoplastic tissues (NS), whereas SUFU expression was downregulated in GC. Thus, the expression levels of miRNA-150 and SUFU are negatively associated with each other. We found that miRNA-150 promoted GC cell proliferation, migration, and EMT and activated the Hh and Wnt/β-catenin signaling pathways by suppressing SUFU expression. Overall, our findings suggest that SUFU mediates the carcinogenic role of miRNA-150. In conclusion, we demonstrated that miRNA-150 targets the tumor suppressor SUFU and promotes cell proliferation, migration, and EMT by activating the Hh and Wnt/β-catenin signaling pathways in human GC.

## MATERIALS AND METHODS

### GC and normal tissues

A total of 50 pairs of GC tumor tissues and non-neoplastic tissues (NS) were collected from gastric cancer patients. Patients signed the consent forms under a protocol approved by Shenzhen University school of medicine. The tissue biopsies were put in RNAlater after resection and stored in liquid nitrogen until needed.

### Cell culture

The gastric cancer cell lines AGS, MKN28 were from the ATCC and China Infrastructure of Cell line Resources, respectively. The gastric cancer cell lines BGC-823, SGC7901 were purchased from Cell Bank of the Chinese Academy of Sciences (Shanghai, China) and control HFE-145 (human normal gastric epithelial cells) were from Dr. Duane T. Smoot at Meharry Medical College. AGS, MKN28 and SGC7901 cells were maintained in RPMI1640 medium (Thermo Fisher Scientific, USA) with 10% FBS (Gibco). BGC-823 and HFE-145 were grown in DMEM (Hyclone, Logan, Utah) with 10% FBS. All cells were cultured in a 5% CO_2_ incubator at 37° C and they had been passed for less than 30 times.

### RT-PCR and real time quantitative PCR

mirVana RNA isolation kits were used for extraction of RNA from GC tissues or cultured cells (Invitrogen, Carlsbad, CA, USA). RNAs were maintained at -80° C until used. The expression of miRNAs was analyzed using TaqMan MicroRNA Assays kit (Applied Biosystems, USA) according to the manufacturer’s instruction. RNU6B (Applied Biosystems) served as an endogenous control. PrimeScript^™^ RT reagent Kit and SYBR® Green Master Mix (Takara, Dalian) were used to synthesize cDNA and quantify the expression of SUFU. GAPDH served as an endogenous control.

### Transfection of miRNAs and siRNAs

MiRNA-150 inhibitors, mimics and their respective NSCs (Nonspecific Control) were obtained from Dharmacon (Lafayette, CO, USA). Cholesterol-conjugated miRNA-150 inhibitors for animal study and their corresponding NCs and three SUFU siRNAs were obtained from Ribobio (Guangzhou, China). A mixture of three SUFU siRNAs was applied in the experiments which successfully down-regulated SUFU expression [13]. When cells reached a confluence of 30-50%, 60 nM of miRNA-150 mimics/inhibitors, or 60 nM of miRNA-150 inhibitors with mixture of SUFU siRNAs were transfected via Lipofectamine RNAiMAX (Invitrogen). Their respective NCs were used as the negative controls.

### Cell proliferation assay

CCK-8 assay (Dojindo, Japan) was performed to assess cell proliferation ability. Cells were first transfected with miRNAs or siRNAs and respective NCs, 48 hrs later cells were collected and inoculated at 1000 cells/well into a 96-well plate. The absorbance at 450 nm was measured by a microplate reader (Molecular Devices, USA) at every other day for 7 days after cultured with 10μl of CCK-8 solution for 1hourat 37° C.

### Cell migration assay

Boyden chambers (BD Biosciences, St Louis, MO, USA) were used to determine cell migration, as described previously [13]. Forty-eight hours after transfection, 5x10^4^ cells were then transferred to the upper Transwell chamber with serum-free medium. The lower Transwell chamber was added with 20%FBS medium as a chemoattractant. After 24 hrs incubation at 37° C, cells that migrated through the membranes were fixed with 4% paraformaldehyde (Solarbio, Beijing) for 15-20 mins followed by hematoxylin staining for 10 mins. After washing with PBS, cells migrating through the pores of the membrane were pictured under a microscope and calculated in five random fields.

### Scratch assay

The scratch assay was performed to evaluate migratory ability. Briefly, 30-50% confluent cells were inoculated into a six-well plate and transfected with miRNAs or siRNAs. Forty-eight hours later when cells reach a confluence of 80-90%, a linear scratch was made using a micropipette tip. The wound closure process was observed and migration gap was photographed using Olympus 1X71 camera system at 0, 24 hours, 48 hours post scratching.

### Western analysis

Laemmli sample buffer (Bio-Rad) was used to extract total protein from cells with a protease inhibitor (Roche) and protein concentration was measured by BCA Protein Assay kits (Pierce, Rockford, MA, USA). Forty-eight hrs after transfection, cells were washed by PBS and incubated with lysis buffer on ice for 30mins. The lysate was then transferred to a tube and the supernatant was collected after 4° C centrifugation. NE-PER™ Nuclear and Cytoplasmic Extraction Kits (Thermo fisher Scientific, MA USA) were used for subcellular fractionation. SDS-PAGE (Bio-Rad) and PVDF membranes (Millipore, Bedford, MA, USA) were used for protein electrophoresis and transfer. The membranes were then blocked and incubated with the primary antibody at 4° C overnight. The membranes were photographed after secondary antibody incubation for 1houron the next day. The primary antibodies were as followed: Wnt/β-Catenin Activated Targets Antibody Sampler Kit (#8655), EMT Antibody Sampler Kit (#9782), SUFU (#2522), Gli1 (#3538) and GAPDH (#5174) (Cell Signaling, Danvers, MA, USA) and Shh (ab53281, Abcam), Smo (ab236465, Abcam), Gli2 (ab187386, Abcam) and Ptch1 (ab53715, Abcam).

### Confocal immunostaining

Forty-eight hours after transfection, cells were permeabilized for 10 mins following fixing with 4% formaldehyde for 20 mins. Cells were blocked with 1%BSA for 1 hour and then incubated with anti-β-catenin antibody (L54E2, 1:100, Cell Signaling) at 4° C overnight. The next day cells were incubated with Alexa Fluor® 594-Conjugated secondary antibody (#8890, 1:1,000, Cell Signaling Technology, Inc.) for 1 hour at room temperature avoiding light. Following immunostaining, DAPI II (Abbott Molecular, Abbott Park, Illinois) was used to stain the nucleus. Images were photographed under a Leica confocal microscope (Cellular Imaging Facility, Lausanne, Switzerland).

### Luciferase reporter assay

TOPFlash and FOPFlash plasmids were purchased from Addgene (Cambridge, MA, USA). Cells were seeded into a 24-well plate and co-transfected with 60 nM of either NSC-inh or miRNA-150 inhibitors and 100ng TOPFlash/FOPFlash plasmids with 10ng pTK-renilla per well via Lipofectamine 3000 (Invitrogen). Forty-eight hours post transfection, cells were then lysed and transferred to an enzyme labeling plate. The Firefly and Renilla luciferase activity were measured by Dual-Glo luciferase assay kit (Promega, Madison, WI, USA).

For SUFU 3’-UTR luciferase assay, the 3’-UTR of SUFU with a predicted binding site of miRNA-150 was subcloned into the reporter vector pDL-UTR (Promega), named pDL-SUFU-3’UTR-wt. Similarly, a mutant-type reporter vector containing three mutated binding sites of miRNA-150, named pDL-SUFU-3’UTR-mut, was also generated as a negative control. Cells were inoculated into a 96-well plate and co-transfected with miRNA-150 mimics/inhibitors and reporter vectors using Lipofectamine 3000. 48 hours post transfection, luciferase activity was measured.

### *In vivo* growth assays

The BALB/c-nu nude mice used in the experiment were all female, 4-6 weeks old and obtained from the Shanghai Laboratory Animal Center (Shanghai, China). All the animal experiments were approved by Institutional Animal Care and Use Committee of Shenzhen University. The animals were free to obtain food and tap water during the experimental periods. Mice were first subcutaneously injected in the flank area with 1×10^6^ BGC-823 cells transfected with either NSC-inh or miRNA-150 inhibitors. Then either NSC-inh (10nmol) or cholesterol-conjugated miRNA-150 inhibitors (Ribobio Co. Guangzhou, China) were injected into the corresponding tumor location twice a week for 37 days (n=6 in each group). Mice were raised in a SPF Laboratory Animal Room with suitable environmental conditions. Tumor length and width were measured twice a week by caliper and tumor volume (mm^3^) was calculated as follows: volume (mm^3^=1/2×length×width^2^).

### ATGC analyses and statistical analyses

The miRNA and SUFU RNA-seq data and patient pathological data were downloaded from The Cancer Genome Atlas (TCGA, https://cancergenome.nih.gov/). We have analysed 368 GCs. We did not analyse the tumor diameter as a parameter, because only 165 samples have this information. The association between pathological characteristics and expression level of miRNA-150 and SUFU was analysed using Kurskal-Wallis test. Overall survival and progression free survival of high and low expression groups was analyzed using Kaplan–Meier survival analysis. Student's t test was used to evaluate the significant difference between the two groups of data. One-way AVONA followed by Dunnett’s test was applied for comparisons in multiple groups. Spearman's correlation was used to analyze the association analysis between miRNA-150 and SUFU. Data is presented as mean ± SD from three independent experiments. A p value less than 0.05 was considered significant.

## RESULTS

### MiRNA-150 targets SUFU mRNA

**Figure 1:**
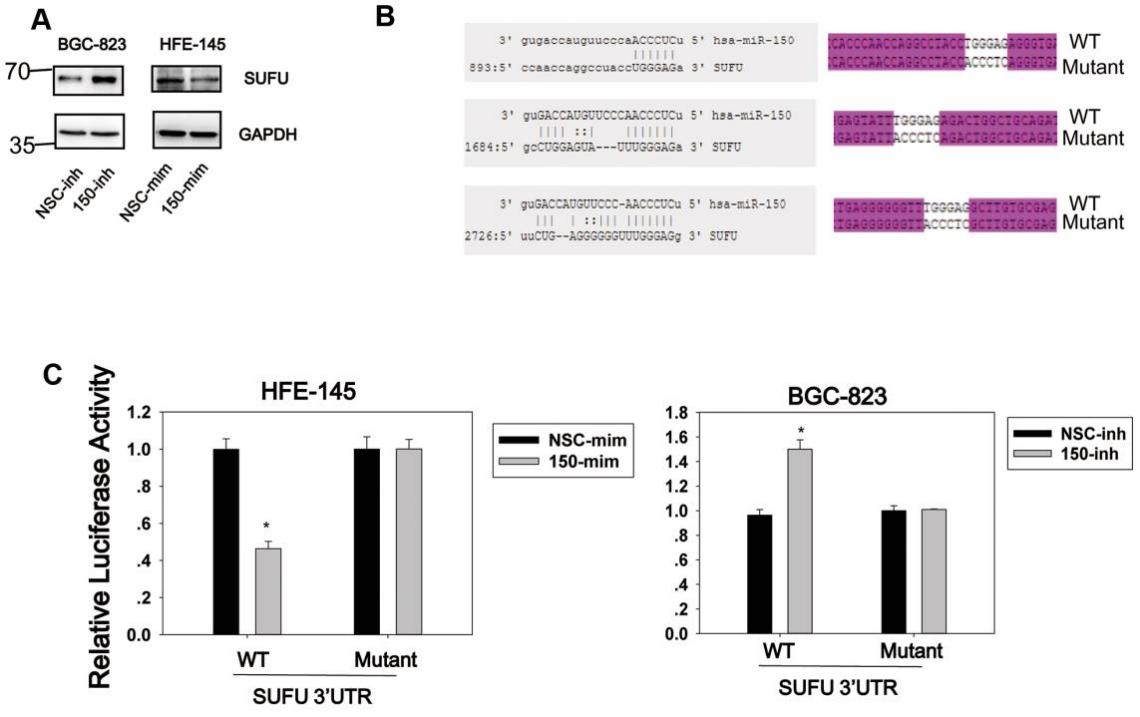
**SUFU is targeted by miRNA-150.** (**A**) The regulation of SUFU by miRNA-150 was verified in HFE-145 and BGC-823 cells by western blotting. (**B**) The binding sites of miRNA-150 on the 3′UTR of SUFU mRNA were predicted. The sequencing result of a vector containing three mutated miRNA-150 binding sites is shown. (**C**) A SUFU 3′UTR luciferase reporter assay was performed in BGC-823 and HFE-145 cells co-transfected with miRNA-150 inhibitors or mimics, respectively, and the SUFU 3′UTR-WT or SUFU 3′UTR-MT reporter.

**Figure 2:**
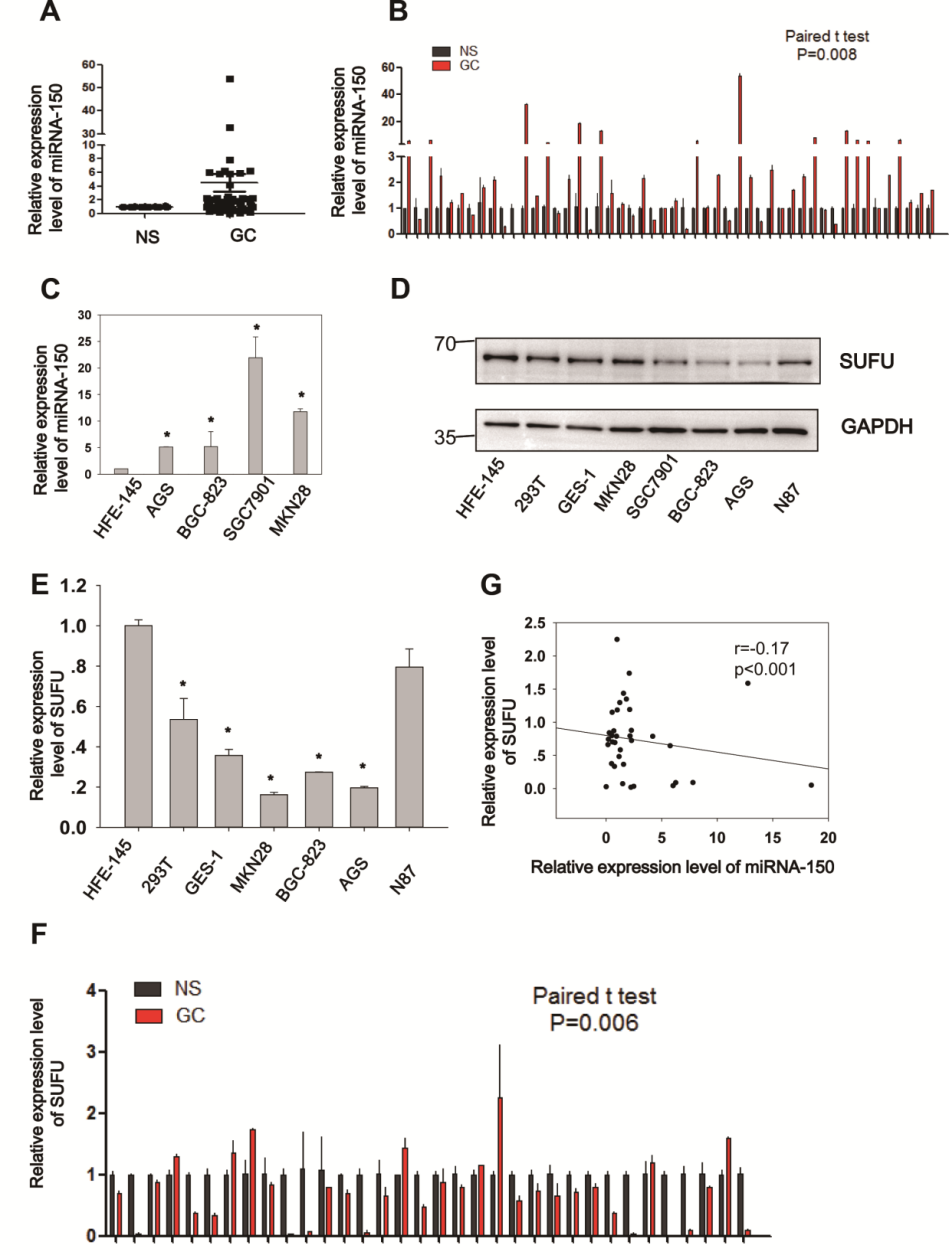
**Negative correlation between miRNA-150 and SUFU in GC.** MiRNA-150 is highly expressed in GC. The expression of SUFU is downregulated in GC and negatively correlated with the expression of miRNA-150. (**A**, **B**) The expression levels of miRNA-150 in 50 paired GC tissues are shown. (**C**) The expression levels of miRNA-150 in GC cell lines, namely BGC-823,AGS,SGC7901,N87 and MKN28 cells and normal epithelial cells (HFE-145, 293T, GES-1) were determined. (**D**, **E**) The expression levels of SUFU in GC cell lines were determined using western blotting and qPCR. (**F**) The expression levels of SUFU in 34 paired GC samples are shown. (**G**) A correlation analysis of miRNA-150 and SUFU mRNA levels was performed in 34 paired GC tissues.

We previously showed that miRNA-194 activates the Wnt/β-catenin signaling pathway by targeting SUFU [13], which acts as a negative regulator in both the Hh and Wnt signaling pathways. Given the importance of SUFU as a tumor suppressor, we investigated whether other miRNAs would also target SUFU mRNA. For this purpose, a library of miRNAs was screened using the SUFU 3′UTR reporter (Supplementary Figure 1), and miRNA-150 was identified as another miRNA that targets SUFU mRNA. As shown in Figure 1A, miRNA-150 mimics (mim) were then transfected into HFE-145 cells, which have a low basal miRNA-150 expression level, and miRNA-150 inhibitors (inh) were transfected into BGC-823 cells, which express high levels of miRNA-150 (Figure 2C). The SUFU expression level was elevated in cells transfected with miRNA-150 inhibitors and decreased in cells transfected with miRNA-150 mimics (Figure 1A). To confirm that miRNA-150 directly binds to the 3′UTR of SUFU mRNA, we analyzed this 3′UTR for miRNA-150-binding sites. We identified three potential miRNA-150-binding sites. A wild-type and a mutant reporter containing three mutated binding sites (Figure 1B) were then constructed and transfected into HFE-145 and BGC-823 cells. In HFE-145 cells, the wild-type reporter, but not the mutant reporter, exhibited significantly reduced luciferase activity with miRNA-150 overexpression. In contrast, in BGC-823 cells, the wild-type reporter displayed increased luciferase activity due to the blocking of miRNA-150 (Figure 1C), indicating that miRNA-150 targets SUFU mRNA specifically by binding to its 3′UTR.

### Negative correlation between miRNA-150 and SUFU in GC

To elucidate the expression and biological role of miRNA-150 in GC, 50 paired GC samples were subjected to qRT-PCR (Figure 2A, 2B). Significantly higher expression levels (Student’s *t*-test, p=0.008) of miRNA-150 were observed in GC samples than in normal samples (NS). Similarly, miRNA-150 was also overexpressed in GC cell lines (AGS, BGC-823, SGC7901, and MKN28) relative to the normal gastric epithelial cell line HFE-145 (Figure 2C, p<0.05). and the AGS, BGC-823, SGC7901, and MKN28 GC cell lines. To further validate the interaction of miRNA-150 with SUFU mRNA, the expression of SUFU was tested in GC cell lines and 34 paired GC samples. Consistent with the previously reported results, both the protein (Figure 2D) and mRNA (Figure 2E) levels of SUFU were downregulated in GC cell lines relative to those in the normal cell line. SUFU expression was also downregulated in the 34 paired GC samples (Figure 2F). Notably, the expression level of miRNA-150 was negatively associated with that of SUFU (Pearson’s correlation, p<0.001, Figure 2G), which further confirmed that miRNA-150 targeted SUFU mRNA in GC. Additionally, an analysis of the pathological characteristics of GC based on the ATGC data showed that the expression levels of SUFU and miRNA-150 are associated with tumor differentiation (Table 1). The data suggest that both are important regulators of GC differentiation.

**Table 1:** The association between pathological characteristics with the expression level of SUFU and miRNA-150.

**Characteristics**		**SUFU expression**		**hsa-miR-150 expression**	
	**No.**	**mean±SD**	***P***	**mean±SD**	***P***
**Differentiation**			**0.0226**		**0.0001104**
High	7	9.047±0.451		9.268±1.268	
Modest	132	8.8±0.493		8.615±1.394	
Poor	220	8.982±0.527		9.205±1.405	
Unknown	9	8.81±0.596		8.065±1.059	
**Distant metastasis**			0.235		0.237
Absent	331	8.924±0.508		8.949±1.39	
Present	19	8.968±0.632		9.626±1.711	
Unknown	18	8.66±0.604		8.608±1.566	
**Lymph node metastasis**			0.628		0.549
N0	113	8.956±0.525		8.902±1.293	
N1	98	8.918±0.526		9±1.508	
N2	74	8.846±0.511		8.81±1.419	
N3	76	8.931±0.521		9.19±1.5	
NX	7	8.78±0.551		8.732±1.581	

*P* values were calculated using the Kurskal-Wallis test; P value < 0.05 in bold.

### MiRNA-150 plays an oncogenic role *in vitro*

**Figure 3:**
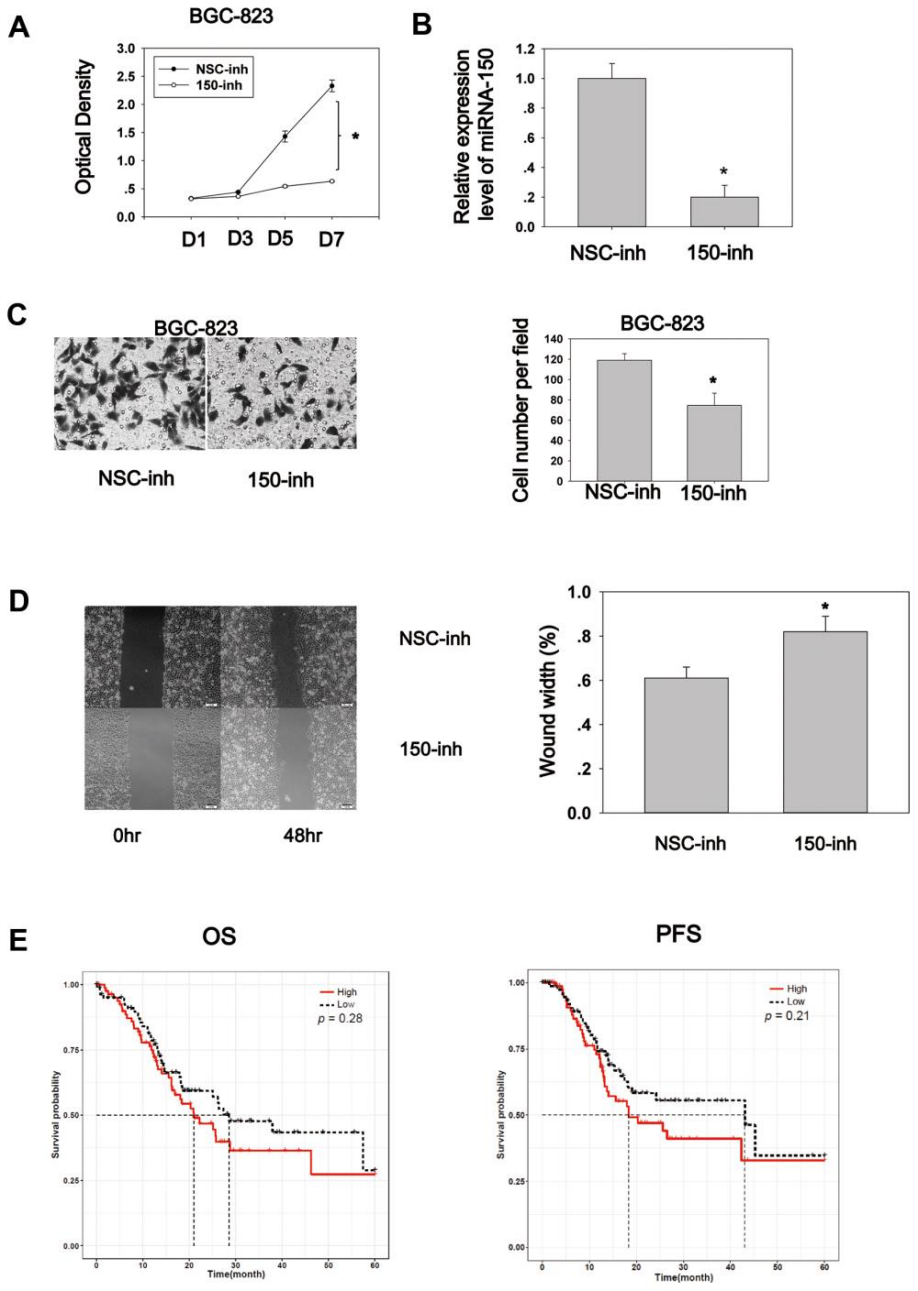
**MiRNA-150 plays an oncogenic role in GC *in vitro*.** (**A**) MiRNA-150 enhanced cell proliferation. Cell proliferation was determined in BGC-823 cells using a CCK8 assay. (**B**) The expression level of miRNA-150 in BGC-823 cells decreased successfully after transfection with miRNA-150 inhibitors. (**C**) MiRNA-150 boosted cell migration. The migratory ability of BGC-823 cells was assessed using a Transwell assay. Five fields in each well were randomly chosen to measure migration. The experiment was repeated three times. (**D**) MiRNA-150 inhibition impaired the migration ability of BGC-823 cells. Five fields in each wound were randomly chosen to measure migration. The experiment was repeated three times. (**E**) Overall survival (OS) and progression-free survival (PFS) in groups with high and low expression of miRNA-150 were analyzed using a Kaplan–Meier survival analysis of the ATGC data of patients with stage III and IV GC. *p<0.05.

To explore the biological role of miRNA-150, cell proliferation and migration assays were conducted. The cell proliferation assay was performed using the CCK8 reagent. BGC-823 cells transfected with miRNA-150 inhibitors exhibited an appreciably lower proliferation rate compared to cells transfected with NSC-inhibitors (Figure 3A). The RT-PCR results showed that the transfection of miRNA-150 inhibitors successfully reduced the expression of miRNA-150 (Figure 3B). Cell migration was also inhibited due to the blocking of miRNA-150 by inhibitors, suggesting that miRNA-150 plays an oncogenic role in gastric carcinogenesis (Figure 3C). Furthermore, a wound healing assay revealed that miRNA-150 inhibition impaired the migration ability of BGC-823 cells (Figure 3D). To study the survival significance of miRNA-150, a Kaplan–Meier survival analysis was performed based on the TCGA data. No significant difference was observed in the survival significance of miRNA-150 across the total patients (Supplementary Figure 2). However, in late-stage (III and IV) patients, high expression of miRNA-150 appeared to be associated with worse overall survival and progression-free survival, although this association was not significant (Figure 3E). This finding suggests that the further subgrouping of staged GC patients would be critical to the use of miRNA-150 as a potential prognostic factor. Evaluations in more precisely defined subpopulations may reveal the true significance of miRNA-150 in GC.

### MiRNA-150 activates the Hh and Wnt signaling pathways and induces EMT

**Figure 4:**
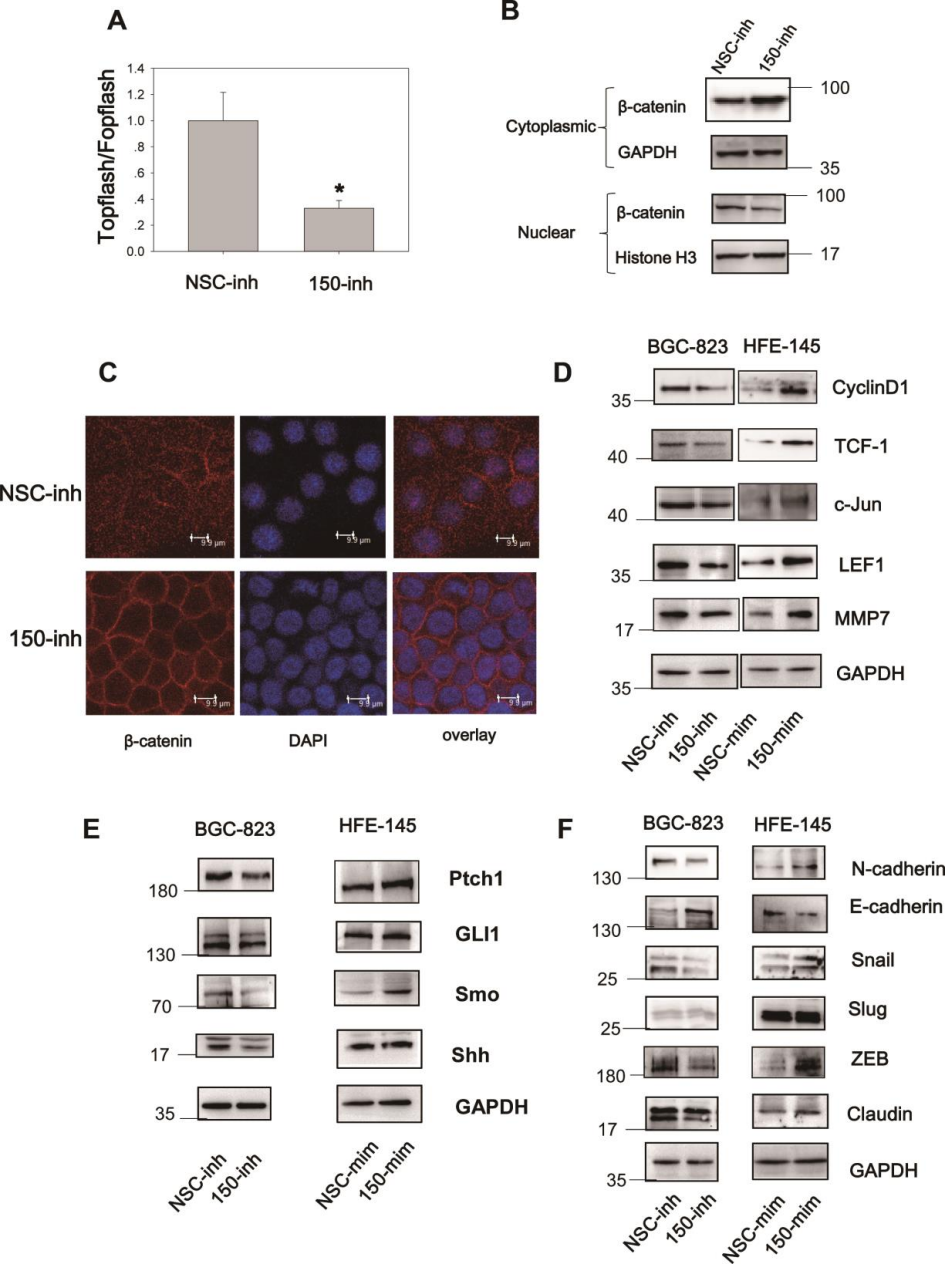
**MiRNA-150 triggers the activation of the Wnt and Hh signaling pathways.** (**A**) MiRNA-150 activated TOPFlash/FOPFlash luciferase activity. A TOPFlash/FOPFlash assay was conducted in BGC-823 cells transfected with miRNA-150 inhibitors. (**B**) The expression of β-catenin in the cytoplasm and nuclei of BGC-823 cells transfected with miRNA-150 inhibitors is shown. (**C**) Immunofluorescence analysis of the localization of β-catenin in BGC-823 cells transfected with miRNA-150 inhibitors is shown. (**D**, **E**) MiRNA-150 activated Wnt and Hh target gene expression. The expression of Wnt and Hh downstream target genes in BGC-823 and HFE-145 cells was detected by western blotting. (**F**) MiRNA-150 induced EMT. The expression of EMT markers in BGC-823 cells or HFE-145 cells was measured by western blotting.

Wnt signaling is constitutively active in 30% of GC cases [7], and Hh signaling is aberrantly active in GC [18]. These findings implicate the importance of both pathways in gastric tumorigenesis and progression. We found that miRNA-194 activates the Wnt signaling pathway by targeting SUFU mRNA, which is also targeted by miRNA-150. This led us to speculate that miRNA-150 is a potential regulator of Wnt/β-catenin signaling. To test this, TOPFlash TCF-dependent luciferase assays were performed. Here, miRNA-150 inhibition led to decreased relative TOPFlash/FOPFlash luciferase activities, implying that miRNA-150 maintains constitutively active β-catenin/TCF-dependent transcriptional activity in GC cells (Figure 4A). Furthermore, because Wnt signaling activation relies on β-catenin nuclear translocation [19], we next examined the subcellular localization of β-catenin. We found that the transfection of miRNA-150 inhibitors led to the decreased nuclear translocation of β-catenin, as revealed by immunoblotting after subcellular fractionation (Figure 4B). Consistent with the immunoblotting data, confocal immunofluorescence staining of β-catenin revealed that the transfection of miRNA-150 inhibitors resulted in the translocation of β-catenin to the plasma membrane (Figure 4C). This phenomenon was consistent with the findings from positive control cells that were treated with XAV-939, a well-known Wnt signaling blocker (Supplementary Figure 3). To validate that miRNA-150 can modulate Wnt signaling, the expression levels of Wnt downstream genes were assessed. Decreased expression of the genes encoding Cyclin D1, TCF-1, c-Jun, LEF1 and MMP was observed in BGC-823 cells transfected with miRNA-150 inhibitors (Figure 4D). Consistently, transfection with miRNA-150 mimics led to increases in the expression of the genes encoding Cyclin D1, TCF-1, c-Jun, LEF1 and MMP in HFE-145 cells. These findings suggest that miRNA-150 induces the activation of the Wnt signaling pathway.

Because SUFU is known to block Hh signaling by binding to Gli [20], we next investigated the Hh signaling pathway. Notably, miRNA-150 suppression decreased the expression of Hh signaling genes such as *Ptch1*, *Gli1*, *Shh*, and *Smo*. Consistently, miRNA-150 overexpression led to the elevated expression of *Ptch1*, *Gli1*, and *Smo*, suggesting that miRNA-150 activates Hh signaling by targeting SUFU mRNA (Figure 4E).

In cancer, EMT is an important step in the process of metastatic transformation and is regulated by the Wnt and Hh pathways. We tested whether a high miRNA-150 expression would triggers EMT and determined that the expression of EMT markers was induced after the transfection of miRNA-150 mimics in HFE-145 cells. This was accompanied by the increased expression of N-cadherin, Snail, and ZEB and decreased expression of E-cadherin. The expression of Slug did not change noticeably and was a good control for differences observed in the expression of other EMT-associated proteins. Consistent with these findings, we further found that EMT was blocked in BGC-823 cells transfected with miRNA-150 inhibitors (Figure 4F). Taken together, these findings indicate that miRNA-150 triggers the activation of the Hh and Wnt signaling pathways and induces EMT.

### MiRNA-150 promotes tumor growth and activates Wnt and Hh signaling *in vivo*

**Figure 5:**
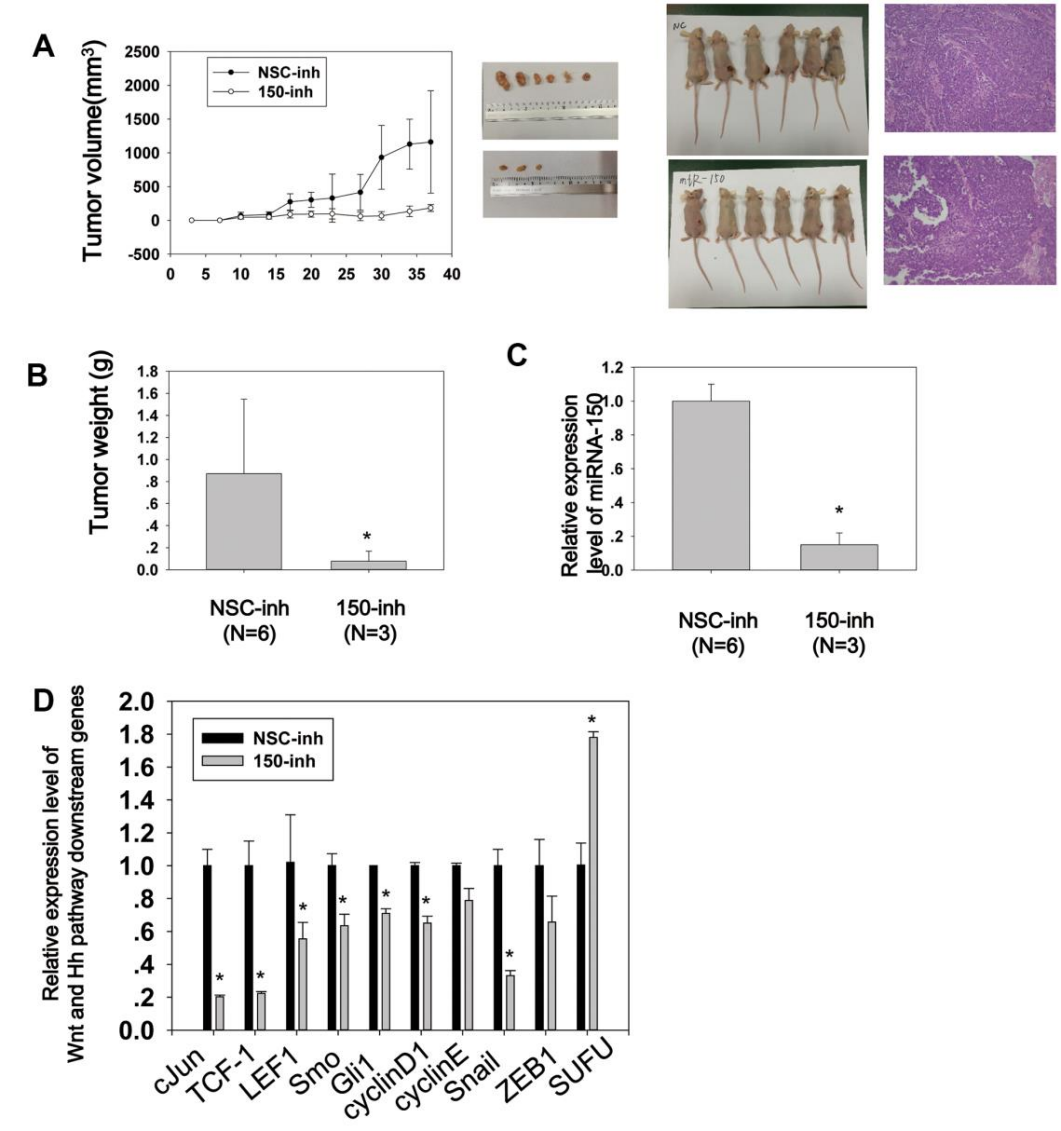
**MiRNA-150 promotes tumor growth and activates Wnt and Hh signaling *in vivo*.** (**A**) Tumor growth curves of volumes measured twice weekly after the injection of miRNA-150 inhibitors. Images of xenograft tumors and nude mice (N=6) and H&E stainings are shown. (**B**) Tumor weights after resection from nude mice. (**C**, **D**) The expression levels of miRNA-150 and Hh and Wnt downstream genes were reduced *in vivo* in tumors treated with miRNA-150 inhibitors. The expression of SUFU was increased in tumors treated with miRNA-150 inhibitors (NSC=6 tumors, 150-inh=3 tumors). Student’s *t*-test was performed. *p<0.05.

To further characterize the biological properties of miRNA-150 *in vivo*, BGC-823 cells transfected with miRNA-150 inhibitors or NSC (non-specific control) were subcutaneously injected into the flanks of nude mice (N=6/group). Tumor nodules in the mice were treated locally with a miRNA-150 inhibitor or cholesterol-conjugated NSC inhibitor suspended in PBS twice weekly until sacrifice. The tumor volume was evaluated before each treatment. As shown in Figure 5A, 5B, the tumor mass volume and weight were significantly less in mice treated with miRNA-150 inhibitors than in control mice (p<0.05). In fact, the tumors became undetectable in three of six treated mice, indicating that the miRNA-150 inhibitors had a good therapeutic effect. Further, we have tried our best to analyze IHC of Wnt and Hh target proteins on these tumor samples. However, the IHC staining was negative in most of the tumors due to long term storage of tumor samples since we have conducted the *in vivo* study in 2016. To evaluate the regulatory role of miRNA-150 in Hh and Wnt signaling *in vivo*, the expression of miRNA-150 and downstream genes in pooled tumor samples was tested using quantitative PCR. The expression of miRNA-150 was substantially lower in the inhibitor-treated tumors than in the NSC-inhibitor-treated tumors (Figure 5C). Furthermore, the expression of downstream genes, including *c-Jun*, *TCF-1*, *LEF1*, *Smo*, *Gli1*, *cyclinD1*, *cyclin E*, *Snail*, and *ZEB1*, was downregulated, whereas that of *SUFU* was elevated (Figure 5D) in the inhibitor-treated tumors. Taken together, these results indicate that miRNA-150 is carcinogenic and activates the Hh and Wnt signaling pathways in GC *in vivo*. Most importantly, the findings demonstrate that miRNA-150 inhibitors have a promising therapeutic effect in GC.

### MiRNA-150 induces Hh and Wnt signaling activation and EMT via SUFU

**Figure 6:**
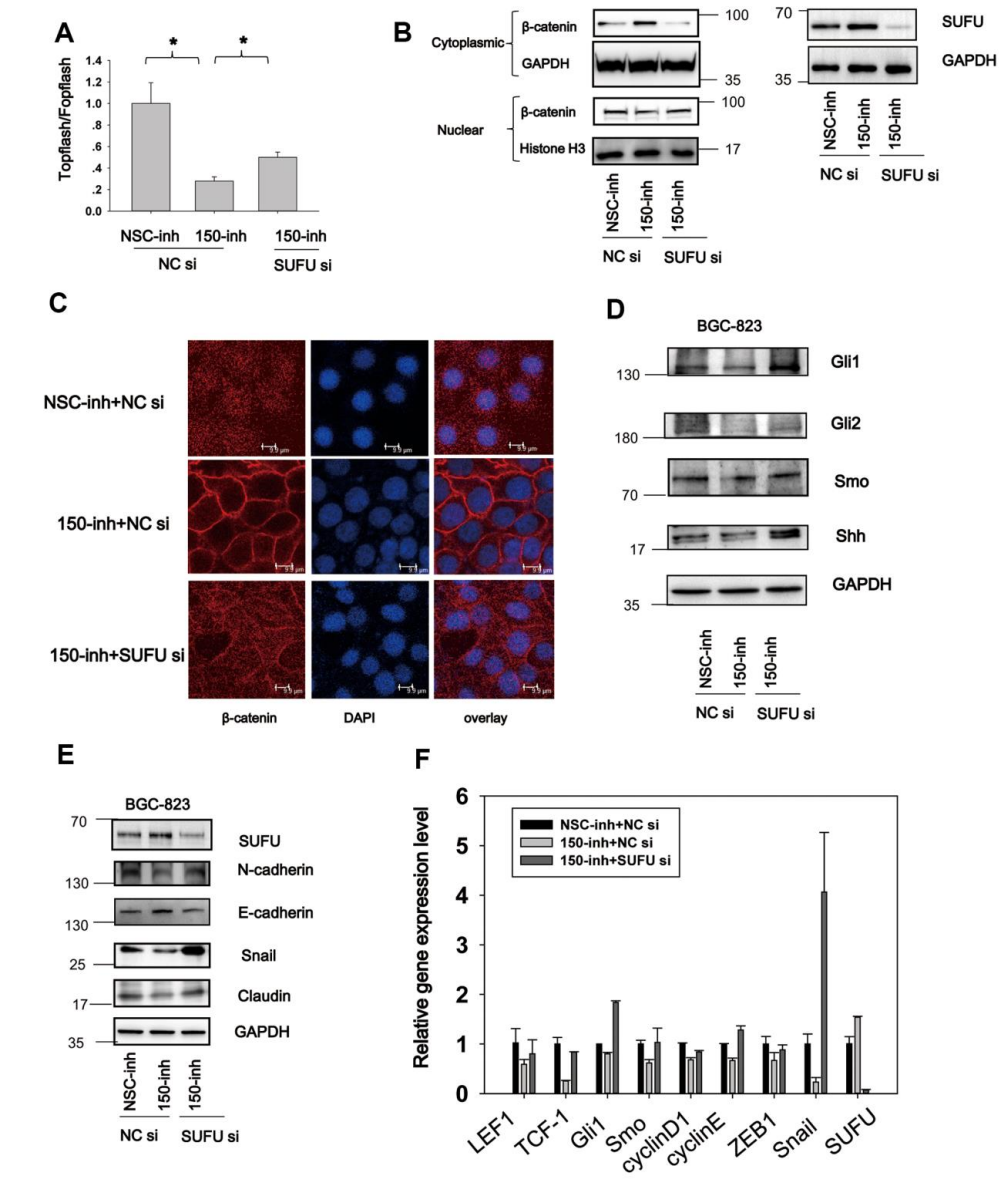
**MiRNA-150 induces Hh and Wnt signaling activation and EMT via SUFU.** (**A**) MiRNA-150 inhibition decreased β-catenin-dependent transcriptional activity, and this decrease was reversed by SUFU siRNAs. A TOPFlash/FOPFlash assay was conducted in BGC-823 cells transfected with miRNA-150 inhibitors or co-transfected with 150-inh and SUFU siRNAs. (**B**, **C**) Interference of SUFU alleviated the β-catenin cytoplasmic translocation induced by miRNA-150 inhibition. (**B**) β-catenin expression was detected in the cytoplasm and nucleus. (**C**) Confocal immunostaining of β-catenin was performed to visualize the cytoplasmic or nuclear localization of β-catenin. (**D**, **E**) MiRNA-150 inhibitors reduced the expression of Hh signaling genes and EMT markers, and SUFU siRNA rescued this inhibitory effect. The expression of Hh signaling and EMT proteins was detected in BGC-823 cells by western blotting after transfection with miRNA-150 inhibitors or co-transfection with 150-inh and SUFU siRNAs. (**F**) The mRNA levels of some Hh-, Wnt- and EMT-related genes were reduced under miRNA-150 inhibition, but increased somewhat after *SUFU* gene knockdown. *p<.05.

The TOPFlash test was performed to determine whether miRNA-150 induces Wnt signaling activation via SUFU. BGC-823 cells were transfected with miRNA-150 inhibitors coupled with SUFU siRNA. As shown in Figure 6A, miRNA-150 inhibitor treatment resulted in decreased TOPFlash/FOPFlash luciferase activity, while SUFU siRNA partly rescued this inhibitory effect (Figure 6A). β-catenin was translocated to the cytoplasm when miRNA-150 was inhibited, whereas SUFU siRNA reversed this translocation (Figure 6B). Confocal immunostaining of β-catenin revealed that this protein was localized preferentially in the cell membrane under miRNA-150 inhibition, whereas SUFU siRNA treatment prevented this localization (Figure 6C). All these findings indicate that SUFU mediates the regulatory effect of miRNA-150 on the Wnt signaling pathway. Meanwhile, immunoblotting to evaluate the expression of Hh signaling genes showed that the Hh signaling inhibition induced by miRNA-150 repression was rescued by SUFU siRNA, suggesting that miRNA-150 modulates the Hh signaling pathway by targeting SUFU mRNA (Figure 6D). To confirm that EMT induction by miRNA-150 was also mediated by SUFU, EMT markers were assessed. EMT was blocked in BGC-823 cells transfected with miRNA-150 inhibitors, and the rescue of this blockade by SUFU siRNA implied that SUFU mediated the induction of EMT by miRNA-150 (Figure 6E). Finally, an evaluation of the expression levels of Hh-, Wnt-, and EMT-related genes by PCR revealed that the expression of these genes was downregulated under miRNA-150 inhibition and restored to normal levels when *SUFU* expression was knocked down (Figure 6F). These data further confirm the mediatory role of SUFU in the induction of Hh and Wnt signaling activation and EMT.

### SUFU mediates the carcinogenic action of miRNA-150

**Figure 7:**
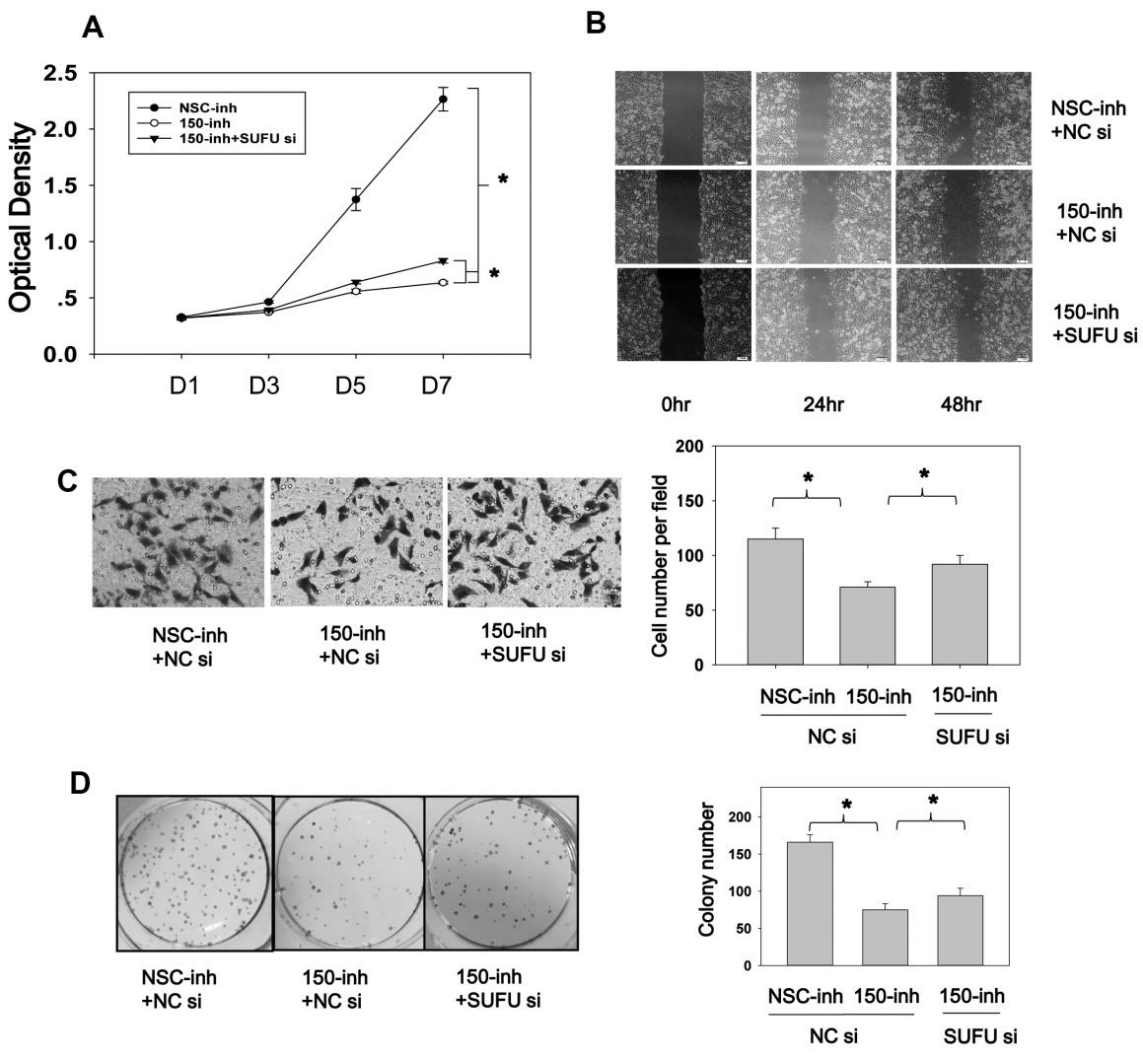
**SUFU mediates the carcinogenic action of miRNA-150.** (**A**) MiRNA-150 inhibition decreased BGC-823 cell proliferation, and SUFU siRNAs rescued this inhibition. Cell proliferation was measured 48 h after transfection with miRNA-150 inhibitors or co-transfection with 150-inh and SUFU siRNAs. (**B**, **C**) MiRNA-150 inhibition suppressed the migratory ability of BGC-823 cells, whereas SUFU siRNAs restored this ability. (**B**) A wound healing assay was performed 48 h after transfection. (**C**) A Transwell assay was conducted 48 h after transfection. Five randomly chosen fields in each well were counted. (**D**) The colony-forming ability of cells was impaired by miRNA-150 inhibitors, while SUFU siRNAs restored this ability. A colony formation assay was performed 48 h after transfection with miRNA-150 inhibitors or co-transfection with 150-inh and SUFU siRNAs. Each experiment was repeated three times. *p<.05.

After determining that miRNA-150 plays an oncogenic role and induces Wnt and Hh pathway activation and EMT via SUFU, we speculated that SUFU is involved as a regulator of the carcinogenic role of miRNA-150. To evaluate this, we performed cell proliferation and migration experiments using SUFU siRNAs. CCK8 assays showed that cell proliferation was inhibited by miRNA-150 inhibitors and that this repression was partly rescued by knocking down the *SUFU* gene (Figure 7A). Cell migration was also suppressed by miRNA-150 inhibitors, whereas SUFU siRNAs partially reversed this inhibitory effect in both scratch (Figure 7B) and Boyden chamber (Figure 7C) assays. It should be noted that the suppression of migration induced by miRNA-150 inhibitors might be due to the inhibition of proliferation. However, SUFU siRNA consistently rescued the inhibitory effects of miRNA-150 inhibitors on proliferation, suggesting that SUFU plays a critical role in mediating the oncogenic function of miRNA-150. MiRNA-150 inhibitors appreciably reduced the colony-forming ability of BGC-823 cells, whereas SUFU siRNAs partially recovered this ability (Figure 7D). Overall, these findings imply that SUFU at least partly mediates the carcinogenic action of miRNA-150 in GC.

## DISCUSSION

**Figure 8:**
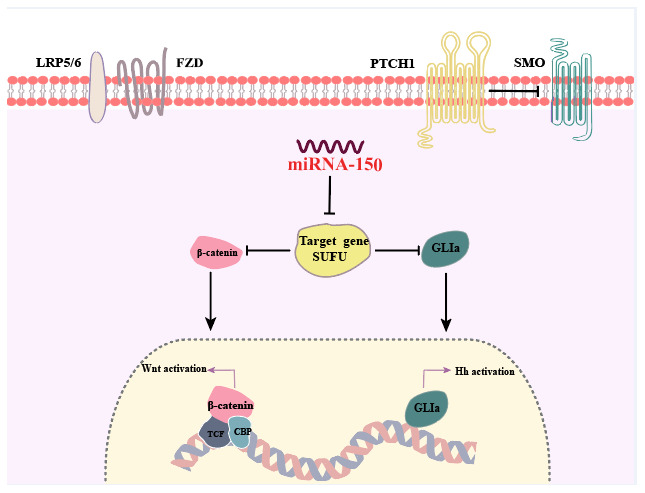
**Summary of the findings.** Dual activation of the Hh and Wnt/β-catenin signaling pathways is induced by miRNA-150 via the downregulation of SUFU expression in human GC.

In this study, SUFU expression was shown to be downregulated in GC tissues that expressed high levels of miRNA-150, and the expression level of miRNA-150 was negatively related with that of SUFU, indicating that miRNA-150 targets SUFU mRNA. We discovered that miRNA-150 is oncogenic and promotes GC cell proliferation, migration, and EMT by activating Hh and Wnt signaling, at least in part, via the suppression of SUFU expression (Figure 8).

Our observation of miRNA-150 overexpression in GC is consistent with the findings reported in the literature. For example, one study reported that miRNA-150 is overexpressed in GC and promotes GC cell proliferation by specifically repressing the expression of early growth response factor 2 [4]. Other studies showed that the higher expression level of miRNA-150 in undifferentiated GC was associated with poorer survival outcomes but was not a significant independent prognostic factor in these patients [21, 22]. In another study that used real-time PCR-based miRNA arrays to test pooled RNA samples from five GC patients, miRNA-150 expression was shown to be upregulated [3]. MiRNA-150 is encoded by a gene located on chromosome 19q13. The amplification of this site has been identified in GC patients [23], which partially explains why miRNA-150 is overexpressed in GC. MiRNA-150 overexpression in GC might also be the consequence of HP infection. HP infection has been strongly implicated in chronic inflammation-induced tumor development. One study showed that HP infection significantly downregulated the expression of MMR genes and upregulated the expression of several miRNAs, including miRNA-150, in AGS cells [6]. In another PCR array-based analysis, five miRNAs, including miRNA-150, were overexpressed in the stomachs of mice with HP-induced mucosa-associated lymphoid tissue lymphoma [24].

We revealed that miRNA-150 acts as an oncogene in GC, but it should be noted that the function of miRNA-150 is highly tissue and micro-environment dependent, as reported in previous studies [25, 26]. MiRNA-150 was shown to be highly expressed in GC, breast cancer, lung cancer, and endometrial cancer and to function as an oncogene in these tumors by repressing the expression of tumor suppressor genes such as *P2X7* and *p53* [26, 27]. In contrast, downregulated miRNA-150 expression has been identified in lymphoma, acute myeloid leukemia, pancreatic cancer, esophageal squamous, colorectal cancer (CRC), and hepatocellular carcinoma, where miRNA-150 targets several oncogenes such as *Akt*, *MUC4*, *ZEB1*, and *c-Myb* [25, 28, 29]. One study of pancreatic cancer determined that miRNA-150 suppresses cell growth and malignant behavior by targeting MUC4 [25]. In contrast, miRNA-150 promotes cell growth and survival in cervical cancer by targeting FOXO4 [30]. Takehiko’s group reported that the expression of the EMT inducer ZEB1 was significantly inhibited by miRNA-150, leading to the induction of mesenchymal–epithelial transition-like changes in esophageal squamous cell carcinoma [29]. Given the distinct functions and expression levels of miRNA-150 in different tissues, the prognostic value of miRNA-150 is expected to vary. A meta-analysis found that miRNA-150 expression was associated with enhanced overall survival in the digestive tract cancer subgroup and poor progression-free survival in various cancers [31]. We found that miRNA-150 is highly expressed in GC clinical tissues as well as cell lines. MiRNA-150 appeared to be a negative prognostic factor in late-stage GC patients, but this association was not statistically significant. We assume that if GC patients were more precisely classified into subgroups, the prognostic significance of miRNA-150 might be clearer.

A single gene can be regulated by multiple miRNAs. Consistently, in a previous study, we found that SUFU mRNA was targeted by miRNA-194 [13]. Although both miRNA-149 and miRNA-150 target the same gene, there are some differences between these microRNAs. First, miRNA-150 has three potential binding sites on the 3′UTR of SUFU mRNA, whereas miRNA-194 has two binding sites, suggesting that the likelihood of binding is higher for miRNA-150. Second, we found that unlike miRNA-150, miRNA-194 did not induce EMT. Third, miRNA-150 inhibition led to dramatic tumor shrinkage, whereas the therapeutic effect of miRNA-194 was only modest. Collectively, these findings suggest that miRNA-150 is an important miRNA in GC that exhibits similarities to and differences from miRNA-194.

SUFU functions as a negative modulator of the Hh and Wnt transduction pathways [32]. It interacts with and retains Gli1 in the cytoplasm and forms a complex with β-catenin to inhibit β-catenin translocation to the nucleus, thus suppressing Wnt signaling [19, 32, 33]. SUFU functions as a tumor suppressor in several malignancies, including lung cancer, prostate cancer, astrocytoma, glioblastoma, and GC [34–38]. In GC, the expression level of SUFU is significantly downregulated and negatively associated with the progression of disease [38]. In our previous study, we found that SUFU expression was downregulated in GC relative to adjacent normal tissues, and that this expression was negatively related to the tumor stage [13, 39]. However, the mechanism underlying this downregulated expression in GC was not clear. In this study, we showed that SUFU is targeted by miRNA-150, which is highly expressed in GC. The expression levels of SUFU and miRNA-150 are negatively associated with each other, which provides another explanation for the aberrant expression of SUFU in GC.

Well-conserved Hh signaling plays a critical role in gastric epithelial cell differentiation and maturation, gastric homeostasis, and malignant transformation. Chronic inflammation caused by HP infection results in poor Shh expression, mostly due to parietal cell atrophy [40, 41]. However, as the disease progresses toward neoplastic transformation, increased Shh expression is observed during epithelial differentiation and replacement. Hh signaling activation is associated with EMT promotion in GC [42]. Gli-1 increases the expression of Snail and consequently decreases that of E-cad [43]. Hh signaling accelerates EMT by upregulating the expression of EMT regulators through the TGF-β pathway [17]. A recent study found that Gli1 expression is negatively associated with E-cad expression and positively associated with VIM expression in GC. Hh signaling inhibition leads to the increased expression of E-cad and decreased expression of VIM, whereas Hh signaling activation has the opposite effects. Overall, these results indicate that Hh signaling activation induces EMT, which in turn enhances GC progression and metastasis [18]. In this study, we found that cells transfected with miRNA-150 mimics exhibited increased activation of the Hh signaling pathway and promotion of EMT, whereas cells transfected with miRNA-150 inhibitors displayed suppression of Hh signaling and inhibition of EMT.

Wnt and EMT are highly associated with tumor development and progression. Accumulating evidence has shown that several miRNAs, including miRNA-150, regulate Wnt signaling activity and EMT by targeting Wnt-related proteins. The positive or negative regulatory effects exerted by miRNA-150 on Wnt signaling and EMT are tumor-type dependent. In some tumor types, miRNA-150 inhibits Wnt signaling and reduces EMT, whereas in others, it activates Wnt signaling and promotes EMT. For example, studies have demonstrated that miRNA-150 targets ZEB1, an EMT inducer, in various cancers such as CRC, epithelial ovarian cancer, and esophageal squamous cell carcinoma. Low expression of miRNA-150 was shown to inhibit cell proliferation, invasion, metastasis, and EMT and to predict a poor prognosis in patients with these cancers [29, 44, 45]. However, miRNA-150 activates Wnt signaling in other cancers. For example, in lung cancer, miRNA-150 was shown to enhance tumor cell metastasis and induce EMT by targeting FOXO4 [30]. In CRC, miRNA-150 enhanced EMT by inhibiting CREB signaling [46]. Furthermore, the Wnt pathway regulates miRNA-150 expression because the promoter region of the gene encoding miRNA-150 contains a potential β-catenin/TCF transcription factor-binding site. The Wnt/β-catenin pathway transactivates miRNA-150 in CRC cells and breast cancer cells [3, 46].

Recent studies have shown the presence of crosstalk between the Hh and Wnt signaling pathways. This crosstalk could serve as a two-way communication with both positive and negative feedback [47]. Our study is the first to show that miRNA-150 activates both the Hh and Wnt pathways by targeting the tumor suppressor SUFU in GC, suggesting another mechanism by which miRNA-150 regulates the Wnt and Hh pathways and the crosstalk between these pathways.

## CONCLUSIONS

In conclusion, we identified significant miRNA-150 overexpression and downregulated SUFU expression in GC and a negative association between the expression levels of these factors. MiRNA-150 activates GC cell proliferation and migration and promotes EMT by targeting SUFU mRNA to activate the Hh and Wnt/β-catenin signaling pathways. This finding provides an insight into the oncogenic effect and underlying mechanism of miRNA-150 and provides a rationale for targeting miRNA-150 as a potential GC treatment in future clinical trials.

### Data availability

The datasets used and analyzed during the current study are available from the corresponding author upon request.

## Supplementary Material

Supplementary Figures
