# Supplementary material for: Dual activation of Hedgehog and Wnt/β-catenin signaling pathway caused by downregulation of SUFU targeted by miRNA-150 in human gastric cancer
Source: Aging (Albany NY). 2021 Apr 12;13(7):10749–69. doi: 10.18632/aging.202895 (PMC8064165; doi:10.18632/aging.202895)
Supplement: Supplementary Figures [file aging-13-202895-s001.pdf]

SUPPLEMENTARY FIGURES

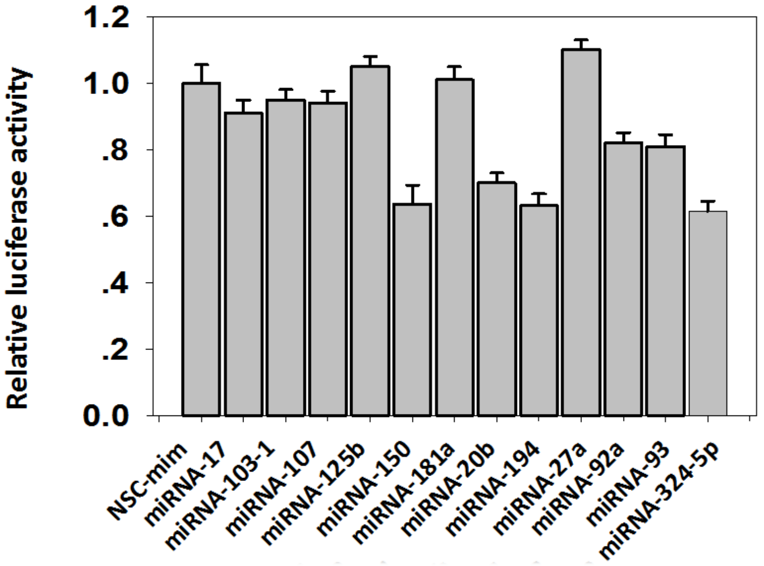

Supplementary Figure 1. MiRNAs which bind and regulate SUFU 3'UTR were screened. A panel of miRNA mimics were transfected into HEK293 cells together with pDL-SUFU-3'UTR plasmid. The relative luciferase activity was determined compared to that in NSC-mim transfected cells.

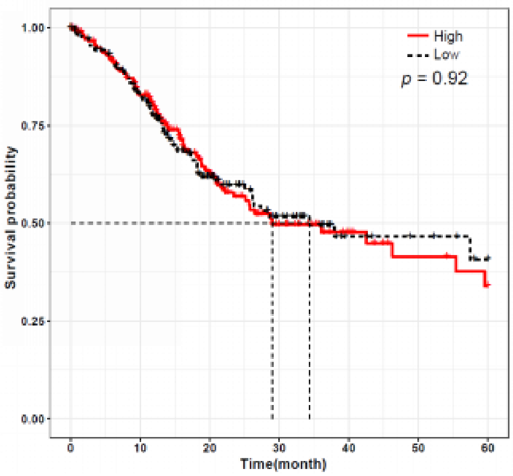

OS

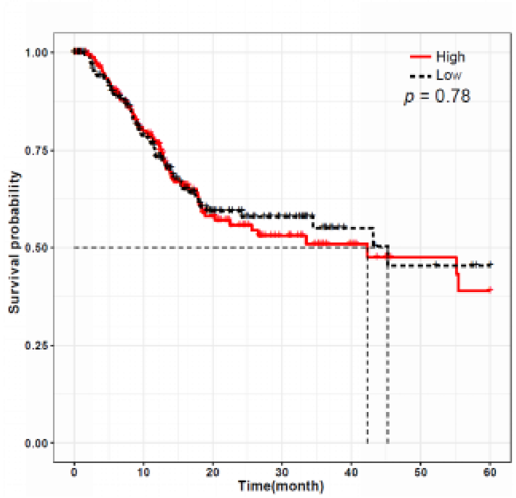

PFS

Supplementary Figure 2. No statistical difference was found for overall survival (OS) or progression free survival (PFS) between patients with high and low expression of miRNA-150.

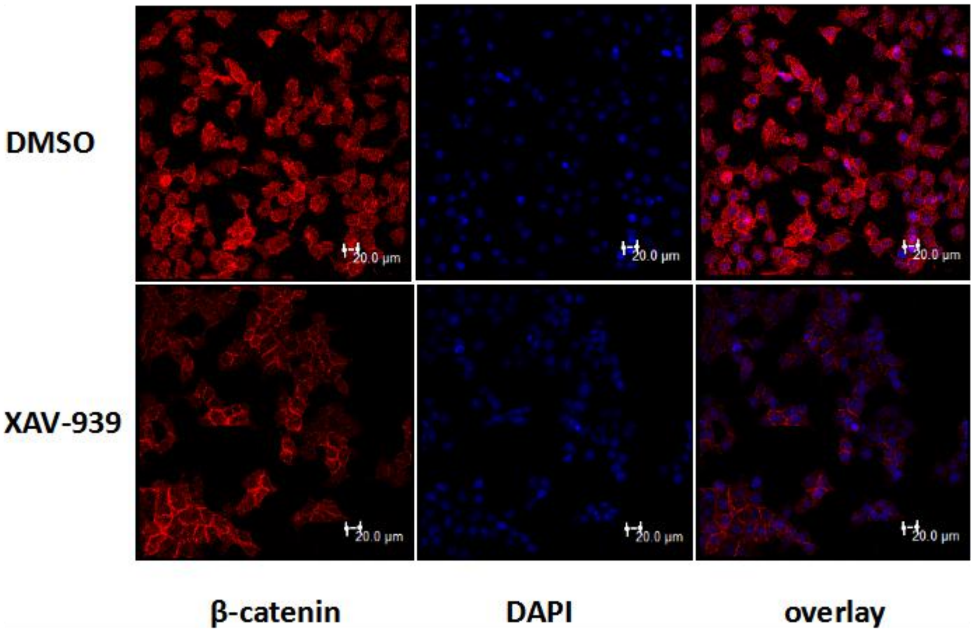

Supplementary Figure 3. Inhibition of Wnt/β-catenin signaling blocked β-catenin translocation into the nucleus. BGC-823 cells were incubated with 1uM XAV-939. Sixteen hrs after drug addition, confocal immunostaining of β-catenin was conducted to detect the β-catenin localization.
